# Marking a New Understanding of Climate and Health

**DOI:** 10.1289/ehp.1611410

**Published:** 2016-04-01

**Authors:** Linda S. Birnbaum, John M. Balbus, Kimberly Thigpen Tart

**Affiliations:** National Institute of Environmental Health Sciences, National Institutes of Health, U.S. Department of Health and Human Services, Research Triangle Park, North Carolina, USA

The month of April brings two observances of significance for many readers of *EHP*: National Public Health Week and Earth Day. The first recognizes the importance of prevention efforts in maintaining the health of our nation’s people; the second, our reliance on and obligation to the health of the planet. This year, April also marks the expected final release of a report that brings the convergence of these two ideas into sharp focus. *The Impacts of Climate Change on Human Health in the United States: A Scientific Assessment* (http://www.globalchange.gov/health-assessment), or Climate and Health Assessment, marks a leap forward in our understanding of the public health implications of climate change.

The report, developed by the U.S. Global Change Research Program as part of its sustained National Climate Assessment process, is the first major U.S. assessment of the scientific literature on climate change and human health since 2008. The assessment breaks new ground by providing quantitative projections of the influence of climate change on five different environmental public health problems, including extreme heat, air pollution, food- and water-related illness and safety, and vectorborne disease. The report also expands a critical discussion of the mental health implications of climate change, and greatly broadens consideration of the issues facing especially vulnerable populations, such as children, the elderly, and the socioeconomically disadvantaged.

Assembled by more than 100 experts over a nearly two-year period, the assessment is a scientific analysis of nearly all the available peer-reviewed literature on the health impacts of climate change and climate-related exposures, as well as much of the gray literature, published in the last half-decade. This highly influential scientific assessment is bolstered by a transparent vetting process, which included vigorous public comment, a National Research Council review, and clearance by the major federal scientific and public health agencies. Thus, it is the best of what we know about how our health is likely to be impacted by climate change.

But even as the Climate and Health Assessment shows the progress we’ve made in understanding the current and potential health impacts of climate change, it also points to large gaps in knowledge that impede our ability to project—and therefore, to prepare for—future climate change health impacts. Many of these gaps are due to insufficient research on the relationship between climate variability and human diseases. For example, while the report highlights modeling studies of how disease-causing vectors, such as ticks and mosquitoes, and bacteria may be affected by future climate change, we still have only a rudimentary understanding of how behavioral and social determinants of health may interact with these climate-related factors to lead to human disease. This gap in understanding prevents us from being able to identify where, when, and in whom new outbreaks of climate-affected disease are most likely to occur in the United States. It also impairs our ability to make decisions that will protect people’s health in the coming years.

What we do know is that climate change is increasing in significance as a public health stressor (Melillo et al. 2014; USGCRP 2008). It is possible to design and implement interventions to limit the impacts and accompanying human suffering caused by climate change, but only if we make the research investments necessary to improve our understanding of how climate change worsens health and determine the most effective interventions. More targeted research programs like the NIH’s Climate Change and Health: Assessing and Modeling Population Vulnerability to Climate Change (http://grants.nih.gov/grants/guide/pa-files/PAR-10-235.html) are needed to help build the nation’s capacity to conduct these kinds of studies. Because climate change and its impacts vary in different regions of the country due to factors such as geography and demographics, regional centers of excellence in climate change and health would greatly enhance our ability to conduct high-quality interdisciplinary research to inform public health practice at the local and regional levels. Comparable regional centers in climate and earth science are currently supported by the Department of the Interior (https://www.doi.gov/csc) and the National Oceanographic and Atmospheric Administration (http://cpo.noaa.gov/climateprograms/%C2%ADclimateandsocietalinteractions/risaprogram.aspxk).

With the release of the Climate and Health Assessment, the climate change and public health communities will now have a greatly enhanced base of integrated knowledge demonstrating how our environment and our health interact. If acted on in comprehensive and innovative ways, this enhanced understanding can point the way towards helping both our communities and our planet become ever more sustainable and resilient, not just for this month, but for all the Aprils to come.
