# Erratum: “Marking a New Understanding of Climate and Health”

**DOI:** 10.1289/EHP350

**Published:** 2016-06-01

**Authors:** Linda S. Birnbaum, John M. Balbus, Kimberly Thigpen Tart

Environ Health Perspect 124(4):A59 (2016), http://dx.doi.org/10.1289/ehp.1611410

In the original article, the URL for the report titled “The Impacts of Climate Change on Human Health in the United States: A Scientific Assessment” was http://www.globalchange.gov/health-assessment.

In this erratum, the authors provide the URL for the final version of this report, which is available at http://health2016.globalchange.gov.

